# Does regeneration recapitulate phylogeny? Planaria as a model of body-axis specification in ancestral eumetazoa

**DOI:** 10.1080/19420889.2020.1729601

**Published:** 2020-02-18

**Authors:** Chris Fields, Michael Levin

**Affiliations:** a Caunes Minervois, France; b Allen Discovery Center, Tufts University, Medford, MA, USA

**Keywords:** Bioelectricity, BMP pathway, eumetazoa, nervous system, symmetry breaking, whole-body regeneration, Wnt pathway

## Abstract

Metazoan body plans combine well-defined primary, secondary, and in many bilaterians, tertiary body axes with structural asymmetries at multiple scales. Despite decades of study, how axis-defining symmetries and system-defining asymmetries co-emerge during both evolution and development remain open questions. Regeneration studies in asexual planaria have demonstrated an array of viable forms with symmetrized and, in some cases, duplicated body axes. We suggest that such forms may point toward an ancestral eumetazoan form with characteristics of both cnidarians and placazoa.

## Introduction

What is the connection between spatial symmetry breaking and multicellularity? To what extent can an ur-metazoan ancestor by envisaged as an initially adventitious, spherically symmetric aggregation of ancestral unicells, e.g. of choanoflagellates as suggested by the “choanoblastaea” model [1], see also [2,3]? Spatial asymmetries clearly predate multicellularity: the Bacilli and the spiral bacteria are classified by their non-spherically symmetric shapes. Even *E. coli* exhibits substrate-dependent chirality at the colony scale [4]. Unicellular eukaryotes exhibit a vast array of internal and external spatial asymmetries. How are such spatial asymmetries translated to the scale of a multicellular organism, particularly a metazoan with well-defined cell layers and multiple distinct organ systems arranged in a specific, population-invariant pattern? The ability to systematically manipulate body-axis asymmetries during whole-body regeneration (WBR) may provide a route toward answering these questions. Organisms capable of WBR are found in all five primary metazoan clades, including the placozoa [5], sponges [6], and ctenophores [7] as well as bilaterians and cnidarians [8]; hence WBR is widely regarded as an ancestral metazoan trait [8–10]. Here we will focus on WBR outcomes in asexual freshwater planaria (Platyhelminthes, Turbellaria, Tricladida), by far the most extensively manipulated WBR model system [11,12], mentioning supporting results from acoel worms (Xenacoelomorpha) and *Hydra* (Hydrazoa) where available.

Distinct body axes, along which differentiated structures can be asymmetrically arranged, provide the basis for Eumetazoan morphologies. With the advent of whole-genome sequencing and transcriptomics, it has become evident that the eumetazoan sister clades of cnidarians and bilaterians employ homologous “developmental toolkits” for body-axis specification [13–16]. Considerable molecular as well as embryological evidence supports homology between the primary cnidarian aboral – oral (A-O) and bilaterian anterior – posterior (A-P) axes [3,17–19]. While a second, dorsal – ventral (D-V) axis breaking the otherwise cylindrical symmetry around the A-P is a defining bilaterian trait, both molecular and anatomical evidence support a secondary (“directive”) axis in at least some cnidarians [20–23]. A third, left – right (L-R) asymmetry appears in some arthropods (e.g. in lobsters) and is ubiquitous in vertebrates [24,25]. We focus here on the early-appearing A-P and D-V axes and their morphological correlates, particularly the gut and central nervous system (CNS) axes.

While many treatments are known that specifically disrupt axis specification in multicellular systems (e.g. Wnt, BMP, or bioelectrical pathways for the AP, DV, and LR axes; see below), these processes remain difficult to manipulate arbitrarily and with full control with molecular or embryological methods in either cnidarians and bilaterians. It is not, for example, completely clear at the molecular or cellular level how the morphological asymmetries of the CNS or the gut, or the behavioral asymmetry of forward locomotion, are aligned along the A-P axis in bilaterals. Nor is it known, outside of planaria and acoels (see below), whether such morphological or behavioral asymmetries can be selectively reversed, e.g. to produce A-P symmetric nervous systems or guts. Some putatively basal, acoel bilaterians have rudimentary, net-like nervous systems without evident ganglia or nerve cords, while others have more elaborated structures [26–28], suggesting that the correlation between CNS axis and A-P axis is not universal in bilaterians. The vermiform myxozoa, e.g. *Buddenbrockia* [29,30] exhibit forward locomotion driven by coordinated, A-P aligned muscle groups, but are radially symmetric cnidarians that altogether lack nervous systems. While such organisms are morphological outliers and may exhibit substantial derived loss of function, their existence renders reconstruction of ancestral axis-specification mechanisms and, in particular, the morphology and expected behavioral repertoire of the common eumetazoan ancestor less than straightforward even given extensive comparative genomics.

Here we suggest that WBR [8–10] provides a tractable alternative to embryonic development for asking fundamental questions about body-axis specification and deep ancestral morphology. We use the term “WBR” to indicate regeneration of the whole body from non-germ cells following either natural or laboratory-induced injuries. In the asexual freshwater planaria of primary interest here, specific experimental manipulations of WBR can symmetrize the A-P axis, including the nervous system and gut [11], add ectopic A-P axes [31], or remove the A-P axis altogether to produce outcomes radially symmetric around the remaining D-V axis [32]; see also [12] for review and specific details below. These manipulations support a suggested homology between the ventral nerve cord (VNC) of bilaterians and the circumoral nerve ring of cnidarians. We reconstruct a hypothesized ancestral eumetazoan characterized by a D-V axis, a blind gut, a nerve ring with a surrounding nerve net, and asexual reproduction. We suggest that the primary function of the nervous system is this animal was not locomotion or feeding but the regulation of body size and morphology.

## Planaria exemplify basal bilaterian morphology and WBR capability

While the early phylogeny of the Metazoa remains controversial, there is broad agreement across models that the Cnidarians and the Bilaterians are sister clades [15,33]. The early phylogeny of Bilaterians is similarly controversial, with numerous models now recognizing the Acoelamorpha as basal bilaterians [34–38]; see [39] for a discussion of conflicts between molecular and developmental-morphological phylogenetic analyses. These animals are characterized by an unsegmented body plan, blind gut, and in some species, an L-R symmetric, multiple-VNC nervous system [18,40], although as noted above, nervous-system morphology is highly variable [26,28]. The development of robust acoel model systems including *Isodiametra pulchra* [41,42] and *Hofstenia miamia* [43–45] has allowed the biology of these organisms, including their regenerative capabilities, to be characterized. Srivastava et al. [45] showed that *Hofstenia miamia* is capable of WBR mediated by the Wnt and BMP pathways as it is in both planaria and *Hydra* [see also 43]. Regeneration is enabled by somatic stem cells (neoblasts) expressing *piwi* homologs, as it is in WBR-capable planaria [39]. In contrast to *Hofstenia miamia, Isodiametra pulchra* is capable of posterior regeneration, but not WBR [42]. Such variability in WBR capability is also observed in planaria [12].

Despite recent progress with acoels, the asexual planarian model systems *Dugesia japonica* and *Schmidtea mediterranea* remain the best-characterized and most extensively manipulated organisms with which to study WBR. While the rhabditophoran Platyhelminths, which include the planarians, are no longer regarded as a basal taxon, they share many of the morphological characteristics of the acoels, including unsegmented body plan, blind gut, and L-R symmetric, two-VNC nervous system [18]. Whether these morphological commonalities are ancestral or derived in either extant acoels or extant planaria remains unknown. Both acoels and planaria exhibit atypical embryonic development [46,47]; whether these characteristics are ancestral or derived also remains unknown.

As with morphology, basal bilaterian reproductive strategy remains controversial [48,49]. While sexual reproduction far pre-dates multicellularity, obligate sexuality appears to be a multicellular innovation in both animal and plant lineages, consistent with Red Queen type arguments [50]. Demosponges and cnidarians such as *Hydra* exhibit opportunistic sexuality with budding and WBR [51,52], suggesting that obligate sexuality is derived from this more flexible strategy [9,53]. Characterized acoels include male-female and cross-fertilizing hermaphroditic species as well as asexuals that reproduce by budding or fission [40]. Characterized planaria include obligate sexual, opportunistic sexual, or asexual species, with some species alternating between sexual reproduction and parthenogenesis or between sexual and vegetative (fission followed by WBR) reproduction [54]. Asexual planaria can be sexualized by feeding them closely related sexual planaria, suggesting that intercellular morphogen-based signaling promotes or enforces sexuality [55,56], inducing stem-cell lineages that would otherwise reproduce to replicate themselves instead to undergo a stem – germ – stem lineage cycle [53,57].

## Manipulating WBR in planaria

Asexual planaria reproduce by fission transverse to the A-P axis followed by WBR of missing anterior or posterior structures [58]; fission is a size and environmental conditions dependent biomechanical process [59] regulated in part by Wnt and BMP pathways [60]. Experimental transverse amputation of both head and tail produce trunk fragments that regenerate both anterior and posterior structures. While amputation of both head and tail does not occur during reproductive fission in the wild, both it and the other manipulations described below are possible outcomes of predation in the wild and engage the same molecular and bioelectric pathways active in reproductive transverse fission. A large number of molecular, pharmaceutical, and bioelectric manipulations have been shown to disrupt WBR in head, tail, trunk, and smaller fragments [11,61]. It is now well-established that the Wnt pathway implements A-P axis specification [62–65], with either bioelectric asymmetry [66] or morphogen transport to the wound site [65] as initiating events. Elements of the Hedgehog (Hh) pathway regulate Wnt pathway activity in both anterior and posterior compartments [67]. Regeneration of specific anterior structures including brain and eyes also depends on the ERK and FGF pathways [65,68,69]. Molecular manipulations implicate the BMP pathway as specifying the D-V axis [11] as in other bilaterians [14].

**Figure 1.**
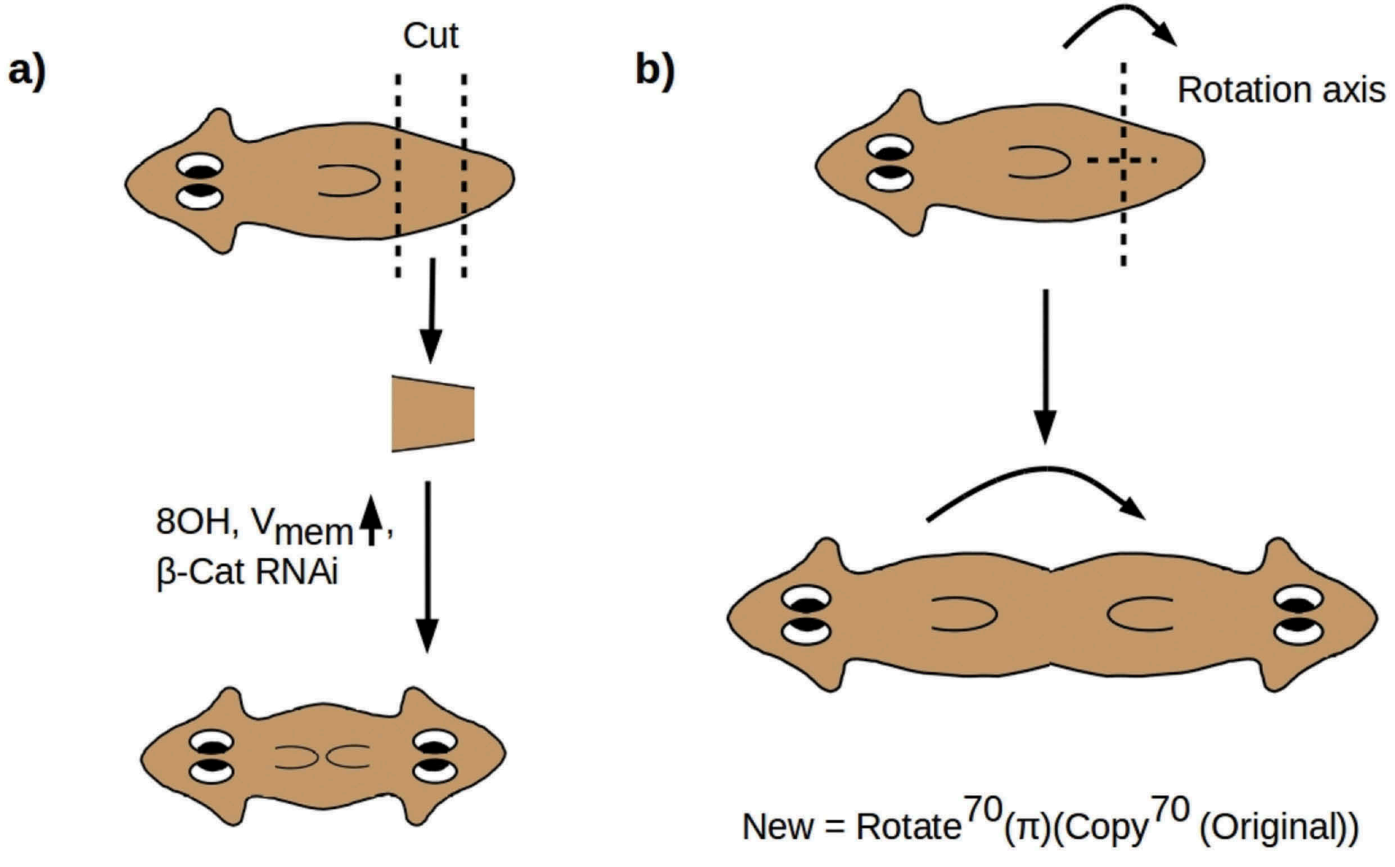
(a) Cutting a PT fragment from a WT worm and treating with 8OH, a depolarizing ionophore, or β-catenin RNAi yields a dose-dependent 2 H phenotype in which all structures anterior to the anterior-facing wound are duplicated. (b) This transformation resymmetrizes the A-P axis around a point at roughly 70% of the worm’s length, equivalent to acting with abstract operators “Copy^70^(π)(•)” and “Rotate^70^(•)” in sequence.

Here we are primarily interested in manipulations that symmetrize the A-P axis, i.e. replace the asymmetric A-P axial morphology with a symmetric A-P-A morphology, or introduce one or more ectopic A-P axes, with radially symmetric forms in which the A-P axis appears to have been altogether eliminated as the limiting case. If a morphologically normal worm is cut at 60% and 80% of its length to make a “pre-tail” (PT) fragment and the fragment is allowed to regenerate, a morphologically normal worm will result. If, however, the PT fragment is treated immediately post-amputation with β-catenin RNAi [70,71], octonol (8OH), a gap-junction blocker [31], or a depolarizing ionophore [66], a two-headed (2H) phenotype results with dose-dependent penetrance [complementary manipulations produce two-tailed phenotypes; see 31,61]. Examination of these 2 H worms reveals that the pharynx has also been duplicated, and the ventral cilia are oriented toward the point of duplication, i.e. in the “posterior” direction from each head [31] as shown in Figure 1a. The nervous system is also duplicated, with both copies functional in directing behavior [72]. Crucially, the VNCs are not only duplicated but are continuous across the duplication point, yielding a nervous system with two complete brains connected by two uninterrupted and apparently fully functional VNCs [31,65,72]. Hence, not only has a head grown from the posterior wound, but the entire anatomy anterior to the anterior-facing wound has been duplicated from the posterior wound. The A-P axis has, in other words, been symmetrized to an A-P-A axis around a point at roughly 70% of the worm’s length, as shown in Figure 1b.

The symmetrization of the A-P axis can be represented geometrically as an abstract rotation by π radians (180°) of a copy of the anterior 70% of the anatomy of the animal (Figure 1b). The axis of rotation is the preserved D-V axis. This “rotation” of the A-P axis is implemented by regenerative growth from the posterior-facing blastema, while regenerative growth from the anterior-facing blastema reproduces the original A-P axis [31,65,72]. Symmetrization of the A-P axis does not erase the distinction between anterior and posterior; it rather duplicates it to produce a bidirectional A-P-A axis with its midpoint at 70% of the original length. The symmetrized animal has duplicated anterior and no posterior anatomy.

Symmetrized 2 H planaria regenerate to produce 2 H progeny for as many generations as have been observed, indicating a stable alteration of morphology. Intriguingly, the morphologically normal outcomes of 8OH treatment under the above conditions are not wild-type, but are rather “cryptic” worms that continue to regenerate 2 H progeny, at the same percentage as in the original experiment, for multiple rounds of regeneration in plain water with no further perturbations [73]. The production of 2 H progeny can be reversed by ionophore treatment, indicating that the “memory” for the 2 H morphology is bioelectric.

*Prima facie* similar axis duplication results have been obtained in acoels [45,74] and Hydra [75]; however, neither multi-generation inheritance of the axis-duplicated phenotype across multiple rounds of regeneration or any analog of the “cryptic” phenotype has been demonstrated in these systems.

**Figure 2.**
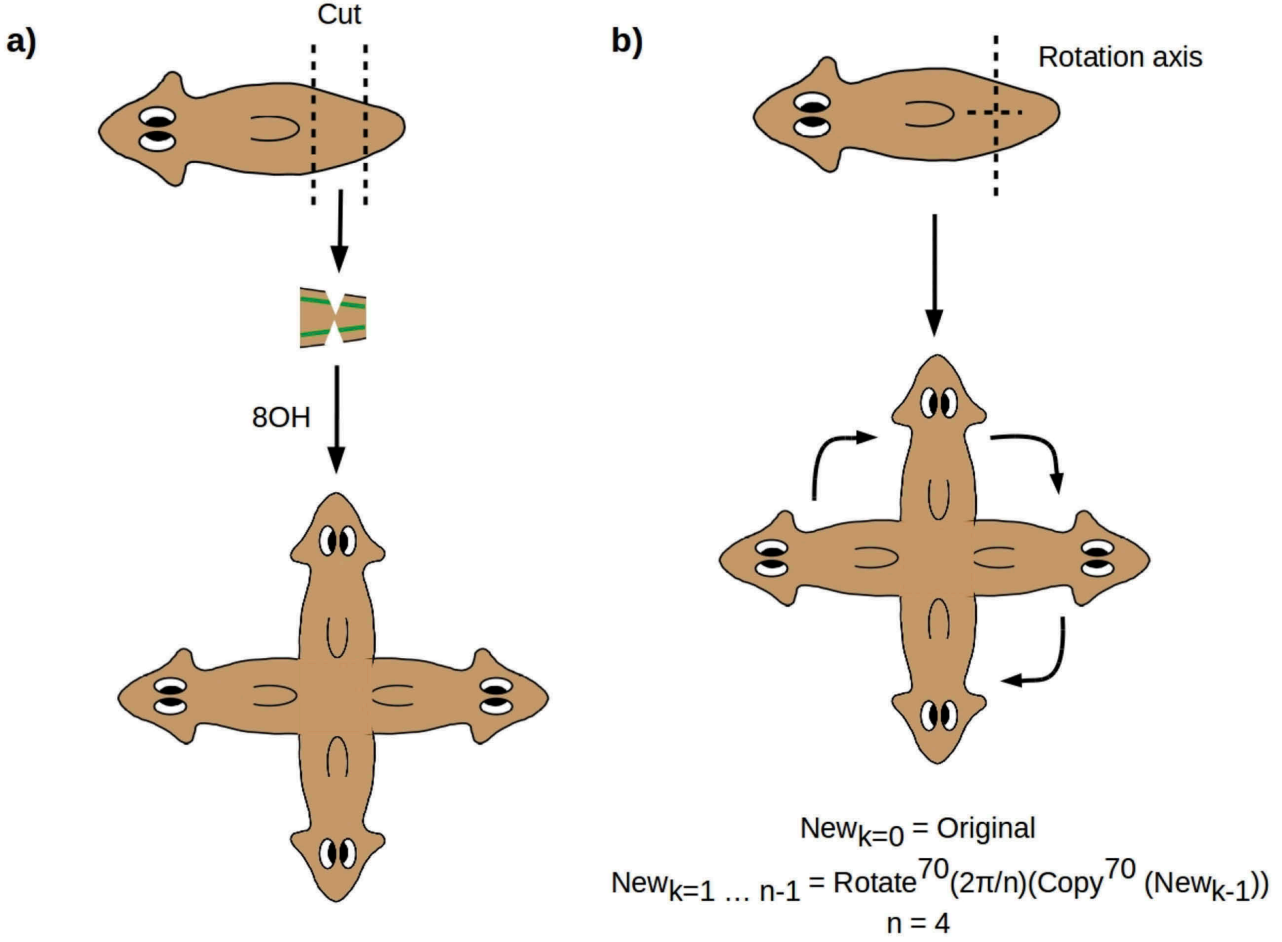
(a) Nicking the two VNCs produces symmetric outcomes with two A-P axes. (b) The outcome can be represented by a repeated copy-and-rotate operation.

Experiments in which the two VNCs are nicked midway through a PT fragment produce symmetric 4 H worms as shown in Figure 2a [31]. Here again, the entire anterior anatomy is regenerated from the two side nicks, producing two symmetrized A-P axes at right angles. As in 2 H animals, the continuity of the VNCs is preserved, with each of the four brains connected by VNCs to the two neighboring brains [31]. This outcome can be represented geometrically as a repeated copy-and-rotate operation as shown in Figure 2b.

**Figure 3.**
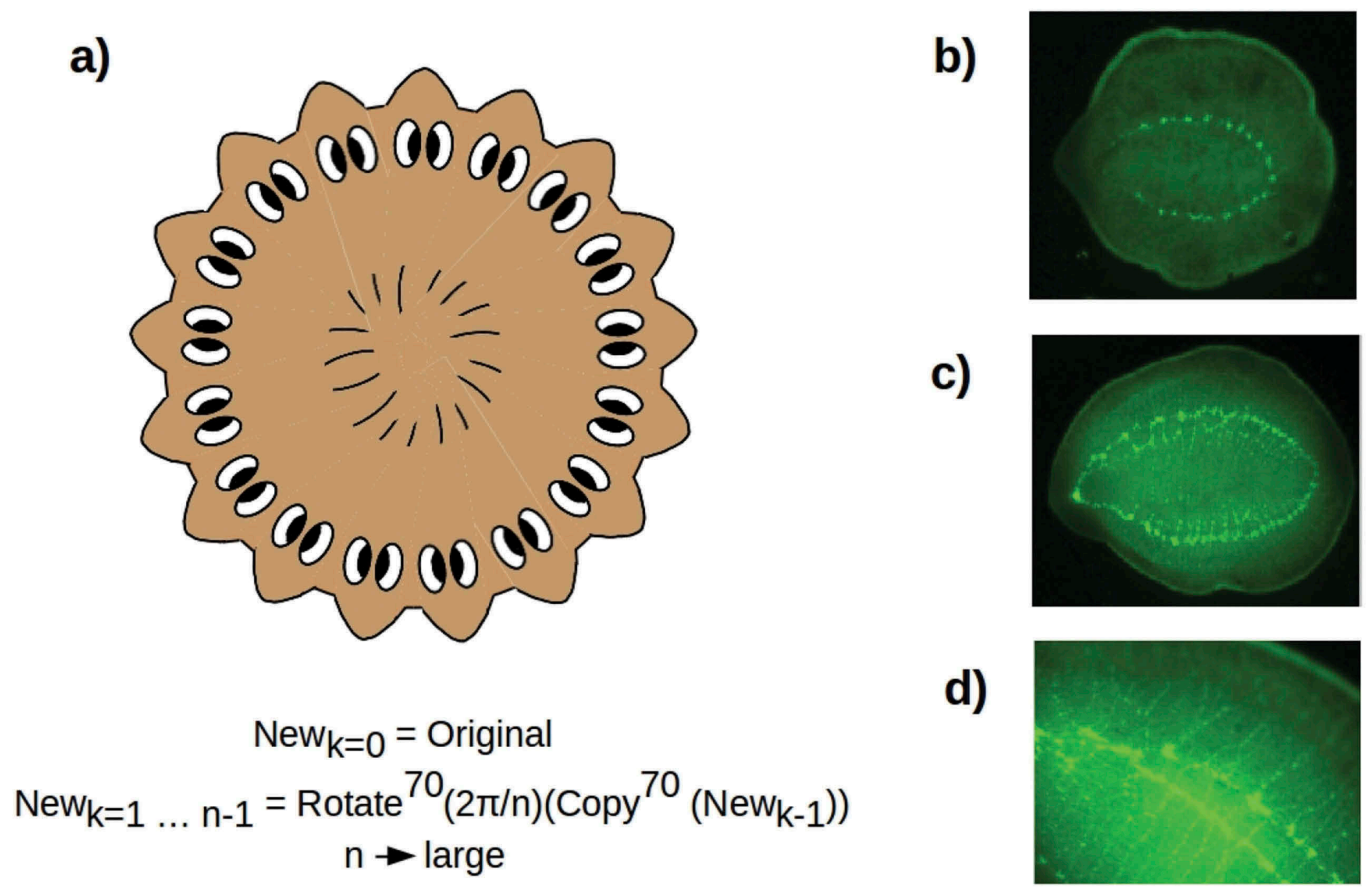
(a) Radially symmetric, hypercephalized outcome of multiple A-P axis duplication and symmetrization as the number n of duplicates becomes large. Such outcomes have been observed following β-catenin RNAi [32]. (b, c) Radially symmetric, hypercephalized outcomes, visualized with synapsin staining, obtained by allowing PT fragments from cryptic worms [73] to regenerate in plain water. d) detail of apparently duplicated circumferential VNC in (c), showing nearly continuous clustering of neurons into apparently proto-cephalic structures [cf. 32, Figure 3].

The effective duplication of a symmetrized A-P axis in the cruciform 4 H animals produced by Oviedo et al. [31] suggests that radially symmetric, hypercephalized outcomes such as sketched in Figure 3a could be produced by making multiple “copies” of the A-P axis and “rotating” them around a central D-V axis. From a geometric point of view, making a large number of copies of the anterior morphology and rotating them in such a way that the heads are evenly spaced is equivalent to simply deleting the A-P axis to produce a radially symmetric, completely anterior morphology. Every radial direction from the central D-V axis is, in this case, “anterior”; hence, any regenerative mechanism that “anteriorized” the animal in a radially symmetric way could be expected to yield this outcome. Such radially symmetric, hypercephalized outcomes were observed by Iglesias et al. [32] up to 4 weeks following β-catenin RNAi treatment of amputation fragments. Consistently with the 2 H and cruciform 4 H regenerates discussed above, these outcomes have a continuous, circumferential “VNC” nerve cord [32]. As predicted, the D-V axis remains unaffected, indicating a lack of significant cross-talk between the A-P (Wnt) and D-V (BMP) axis specification systems. Pharyngeal anatomy is, however, disorganized or lost altogether in these radially symmetric animals, in contrast to its preservation and apparent function in 2 H and 4 H animals, suggesting that radially symmetric anteriorization disorganizes tissue specification near the “origin” of the radial axis. Eyes with optic nerves are present in association with some, but not all, of the apparently proto-cephalic clusters of neurons distributed roughly uniformly along the circumferential “VNC” [32], suggesting some loss of tissue specification distally along the radial axis.

Radially symmetric, hypercephalized outcomes with continuous, circumferential nerve cords (Figure 3b–d) have also been observed following regeneration, in plain water without further perturbation, of PT fragments of cryptic worms [73]. The VNCs appear to be duplicated in some of these regenerates (Figure 3c,d). Neither pharyngeal structures nor eyes are observed in these preparations. These observations suggest that the bioelectric changes that define the cryptic phenotype can have far-reaching and variable consequences for regenerative morphology that await further, detailed investigation.

In summary, the planarian A-P axis appears to be not just symmetrizable, but highly manipulable, in regeneration-based assays. Both molecular (e.g. β-catenin RNAi) and bioelectric (8OH, ionophore) manipulations can lead to axis symmetrization and duplication. The cryptic phenotype identified with 8OH treatment is the first known example of reversible, bioelectric, epigenetic inheritance [73]; a similar reversible bioelectric manipulation has now also been demonstrated in *Hydra* [76]. In the absence of rescue manipulations, the altered phenotypes are stable across multiple generations in viable individual planaria, and may be permanent. These results suggest that while the A-P axis is “primary” in planaria as in other bilaterians, it is in a highly plastic state that may reflect loss of evolved constraints on the standard bilaterian body plan.

## The planarian A-P axis as a transitional state

In cnidarians, Wnt pathway components including the Disheveled (Dsh) receptor and β-catenin effector are expressed in a decreasing gradient from the Oral to the Aboral pole [18,19]. Let us call this the “Wnt – anti-Wnt” axis, where here “anti-Wnt” refers to either a Wnt inhibitor or opposite-pole determinant. The Wnt – anti-Wnt axis is aligned with the gut in cnidarians, orthogonal to the circumoral nerve ring, and aligned with the long axis of the nerve net driving whole-body contractions and motility [77].

In bilaterians, Wnt pathway components are expressed in a decreasing gradient from posterior to anterior. In bilaterians possessing a through-gut, the Wnt – anti-Wnt axis is aligned with the gut and the nerve cord(s) extending from the anterior brain. The anterior, anti-Wnt direction is the direction of both motion and the mouth.

**Figure 4.**
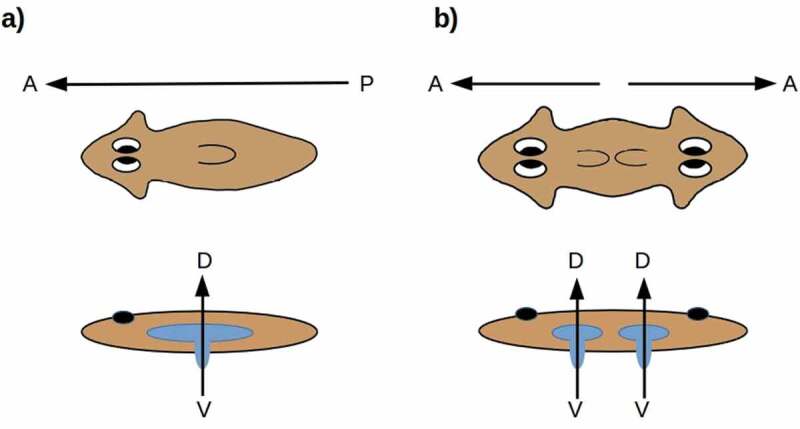
(a) The planarian mouth opening is aligned along the D-V axis, with respect to which the blind gut has the radial symmetry of the blind gut in cnidarians. (b) Symmetrizing the A-P axis duplicates the mouth-opening axis while preserving its D-V orientation.

The structure of the pharynx and hence the location and orientation of the combined mouth/anus is variable in both acoels and flatworms. In the planaria of interest here, the mouth opens ventrally, aligned with the D-V axis [78], along which the pharynx can also be extended as sketched in Figure 4a. The planarian blind gut has the orientation with respect to the D-V axis that the cnidarian blind gut has with respect to the O-A axis. Symmetrizing the A-P axis in planaria duplicates the mouth and pharynx while maintaining their D-V orientation (Figure 4b). In the radially symmetric forms shown in Figure 3, the D-V axis has become the “primary” body axis about which anatomical structures are radially symmetric; the radial symmetry in this case is analogous to the radial symmetry of cnidarians around the O-A axis.

From this perspective, the idea of a “primary axis” appears somewhat ambiguous in planaria. Known manipulations of the D-V axis, moreover, are neither as thorough-going or as extensive as the A-P manipulations reviewed here [11]; no D-V analogs of the multiple A-P duplications shown in Figure 2 or 3 are known. Topologically, the planarian A-P axis is analogous to the cnidarian directive (secondary) axis; each specifies two opposing “sides” of a central, invaginated body cavity. Could the plasticity of the planarian A-P axis reflect an ancestral state in which this axis was secondary, as the directive axis is in cnidarians?

## Reconstructing an ancestral eumetazoan

Deep metazoan phylogeny remains highly controversial, with active disagreement about whether porifera or ctenophores are more basal and considerable uncertainty about the placement of placazoa [79–81]. All empirical phylogeny, however, equally suffers from the problem that only extant (or well-preserved fossil) species are accessible for analysis. An empirically informed theoretical phylogeny may therefore have value in considering questions of eumetazoan ancestry.

**Figure 5.**
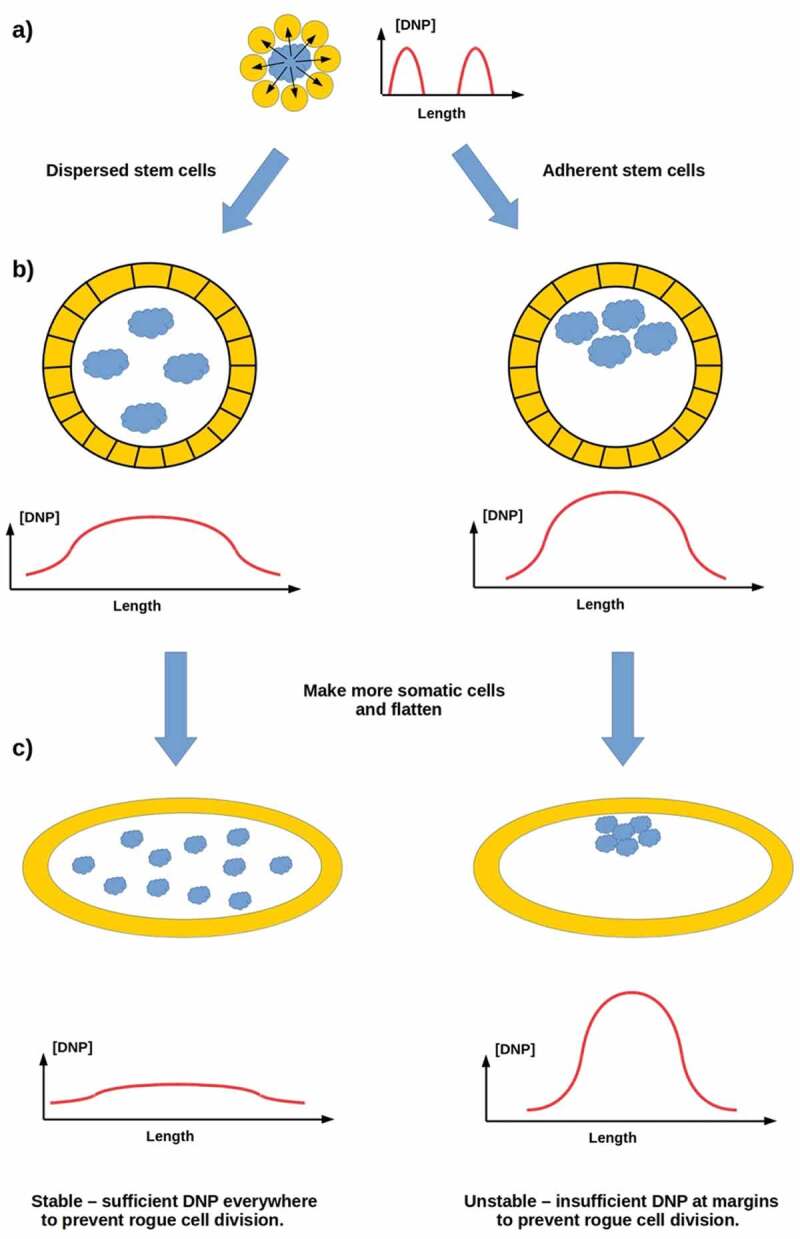
(a) An ancestral proliferative cell produces progeny for protection, employing a short-range “do not proliferate” (DNP) signal to suppress their proliferation. (b) A somatic cell layer enclosing dispersed proliferate cells has uniform [DNP]; if proliferative cells cluster, [DNP] is non-uniform. (c) A primitive organism comprising a cell layer enclosing dispersed proliferative cells is stable; one enclosing clustered proliferative cell has insufficient [DNP] to prevent rogue proliferation at its margins, so is not stable.

Standard models of the emergence of animal multicellularity are based on the aggregation of closely related cells [82], typically choanoflagellates [1–3]. We have recently proposed an alternative, non-aggregative model in which ancestral, free-living stem cells produce a protective “body” comprising their own reproductively disabled progeny as a means of self-defense in a challenging environment [83]. The principal regulator in this scenario is a “do not proliferate” (DNP) signal that the parent stem cell employs to shut down proliferative capability in its progeny, rendering them fully “somatic” cells with no independent genetic interests or fitness. As Wnt-pathway components are already used for proliferation suppression of prestalk cells in *Dictyostelium* [84,85], it is plausible on phylogenetic grounds that this DNP signal may be a Wnt or Wnt analog. The DNP signal is assumed to be secreted only by proliferative stem cells and to be short-range; hence its distribution within a multicellular system will depend on whether the system’s proliferative cells are dispersed or clustered, as sketched in Figure 5a,b. A primitive organism comprising a cellular envelope enclosing a uniform distribution of dispersed proliferative cells may be expected, assuming cell-cycle synchronization or some other mechanism to coordinate stem-cell proliferation, to have relatively uniform internal [DNP] and to be stable. However, such an organism with clustered proliferative cells is expected to have non-uniform internal [DNP] and to be unstable due to uncontrolled reproduction by “somatic” cells in which independent proliferation has not been fully suppressed (Figure 5c).

**Figure 6.**
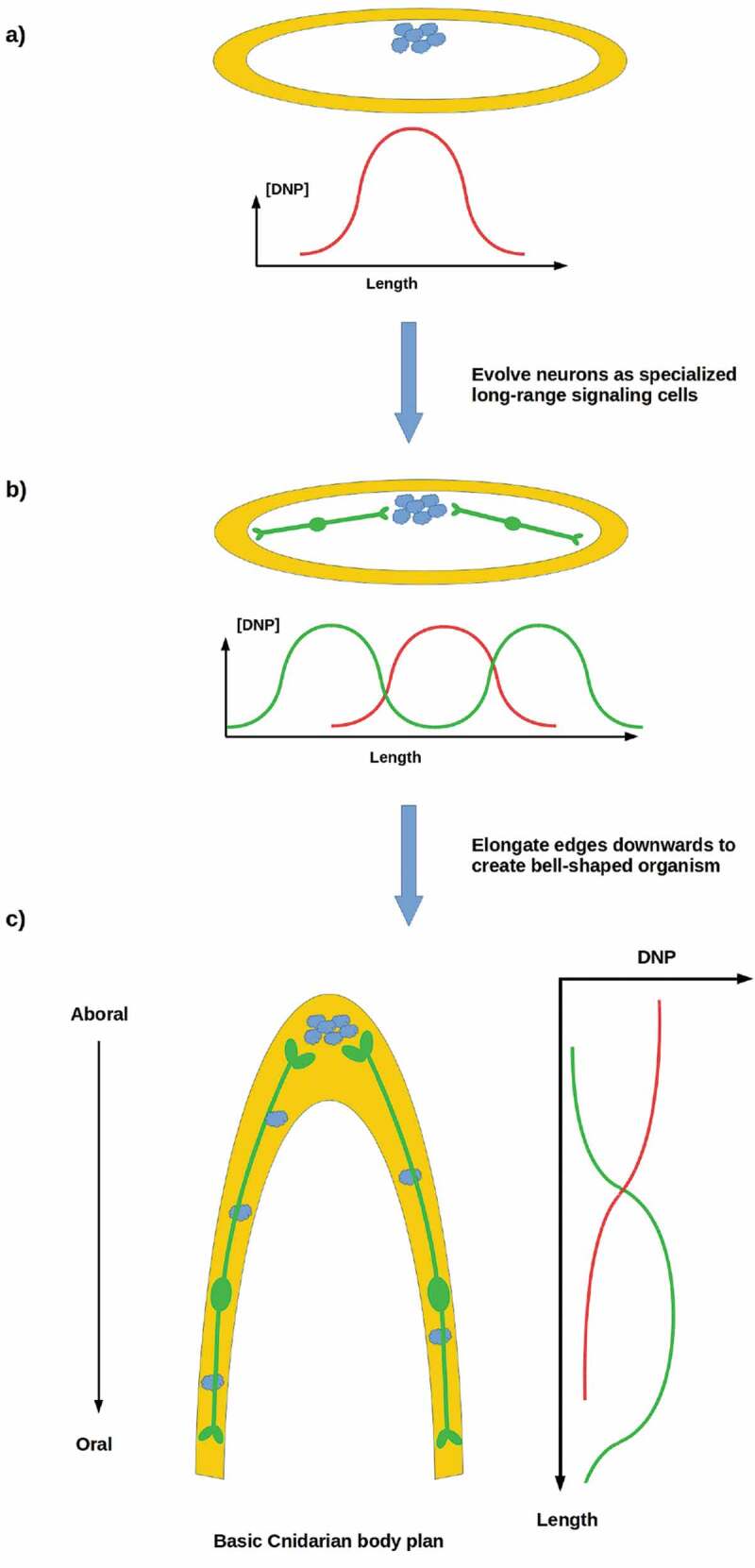
(a, b) A reproductively unstable system can achieve stability by employing neurons to transmit a DNP-like signal (green curves) to distant somatic cells in order to suppress rogue cell division. (c) Neurons enable the development of complex anatomies, e.g. invaginated body cavities.

Reproductive stability is possible with clustered proliferative cells if longer-range communication of a DNP-like signal that suppresses rogue cell division by somatic cells is possible. Neurons provide an ideal solution to this problem, as they allow long-range, error-correcting communication between source cells and specific target cells [86]. Neurons likewise provide a means of coordinating the proliferation of dispersed populations of stem cells that are too far apart or too distant within a cell lineage to be reproductively coordinated by other mechanisms. An organism with neurons can adopt a more complex body plan, e.g. by elongating its periphery into an invagination as sketched in Figure 6. The three extant animal lineages with complex body plans – the ctenophores, cnidarians, and bilaterians – all have neurons. Structural, biochemical, and molecular differences between ctenophore neurons and those of cnidarians and bilaterians suggest convergent evolution to a common function [87]. We have suggested that the primary ancestral function of neurons in all three lineages is the long-distance control of cell proliferation that enables a stable multicellular morphology even with clustered stem cells [86]. While this hypothesis remains to be tested, manipulations in *Xenopus* embryos provide initial evidence for CNS regulation of distal morphogenesis [88,89].

These theoretical considerations suggest an interpretation of the radially symmetric, hypercephalized regeneration outcomes shown in Figure 3 as regressions toward an ancestral state, one that may pre-date not only planaria but eumetazoa in general. Only one extant animal lineage comprises flat, approximately radially symmetric organisms: the placozoa [5,90]. Placozoa have no differentiated neurons but have neurotransmitters and behavior. The dispersed, interior “fiber” cells of placozoa are extended and may serve a communicative function, e.g. by paracrine signaling as in sponges [91]. Characterized placozoa do not have differentiated mouths or guts, but appear to digest food externally and absorb nutrients through distributed ventral-surface cells [5]. Placozoa are predominately asexual, reproducing by budding or fission with WBR, but also exhibit opportunistic sexuality. They are often regarded as amorphous but have a primary D-V axis, the axis normal to the substrate, around which they are approximately radially symmetric. Their anatomy resembles the left side of Figure 5c.

Do the radially symmetric, gutless, hypercephalized regeneration outcomes in Figure 3b, c resemble placozoa with neurons added? While gross morphology suggests that placozoa may be their closest affinity, further investigation of both parties is clearly required to answer this question. If these planarian regenerates indeed resemble placozoa at more than a superficial level, they may be pointing toward an ancestral eumetazoan with radial symmetry around a primary D-V axis, a blind or undifferentiated gut, a rudimentary circumferential nerve cord with radial branching, and asexual reproduction with WBR.

## Conclusion

We have suggested here that WBR provides an alternative to embryology for studying the mechanisms of body-axis specification and their contributions to the evolution of complex morphologies. Asexual planaria appear to be particularly attractive model systems in this regard. The A-P axis of planaria, in particular, is highly malleable using molecular, pharmacological, and bioelectric manipulations. This axis can not only be symmetrized but also duplicated to such an extent that it effectively disappears, leaving a radially symmetric, fully anteriorized form. Whether the A-P axis of acoels, or of other bilaterians, is similarly manipulable remains to be seen; commonalities in axis-specification mechanisms across the bilaterians as well as specific results in acoels [45,74] suggest that they may be.

The evolutionary emergence of complex body plans appears intimately connected to the emergence of neurons as specialized long-range signaling systems [86]. We suggest that the emergence of neurons in a radially symmetric, placozoan-like animal may have set the stage for the differentiation of the eumetazoan lineages.

Further work is clearly required to elaborate and test these hypotheses. Life-history studies of the symmetrized forms shown in Figure 3b, c have already been initiated; if such forms can be reliably produced and maintained, functional investigation of their nervous and digestive systems will be possible. The results of Müller [75] and Braun and Ori [76] suggest that *Hydra* may be an attractive Cnidarian model system with which to pursue similar axis-symmetrization studies. Thorough investigation of cell-cell signaling mechanisms in Placozoa would complement these investigations. More broadly, the study of the regulation of morphogenesis outside of the nervous system *per se* by neural activity remains in its infancy. Recent as well as classical evidence of regulation of regeneration [92] and of transformation and tumor growth by the nervous system [93–95] suggests that such studies may also have significant clinical relevance.
